# Supplementary material for: The Arabidopsis HY2 Gene Acts as a Positive Regulator of NaCl Signaling during Seed Germination
Source: Int J Mol Sci. 2021 Aug 20;22(16):9009. doi: 10.3390/ijms22169009 (PMC8396667; doi:10.3390/ijms22169009)
Supplement: Supplementary file 1 [file ijms-22-09009-s001.zip › Supplemental Table S10.pdf]

Supplemental Table S10: Primer sequence

CDS-*HY2*-F: GGGCGGAAAGGAATTCATGGCTTTATCAATGGAGTTTGG
CDS-*HY2*-R: CGGTGGATCCAAGCTTGCCGATAAATTGTCCTGTTA
hy2-LP: CGCAGAGAACAAAGCCTTATG
hy2-RP: AGAGAGTGTCCGAGGAAGGAG
LB1.3: ATTTTGCCGATTTCGGAAC
RT-*HY2*- F: ACATAGTTGTATTGGACCTT
RT-*HY2*-R: TACTTCGTTTATCGCTTAC
RT-*Actin*-F: AGGCACCTCTTAACCCTAAAGC
RT-*Actin*- R: GGACAACGGAATCTCTCAGC
ACT2-F: TTGTTTGTTTCATTTCCCTTTTTG
ACT2-R: GCAGACGTAAGTAAAAACCCAGAGA
RD29A-F: GGCGTAACAGGTAAACCTAGAG
RD29A-R: TCCGATGTAAACGTCGTCC
RD29B-F: GCGC ACCAGTGTATGAATCCTC
RD29B-R: TGTGGTCAGAAGACACGACAGG
DREB2A-F: AAGGTAAAGGAGGACCAGAG
DREB2A-R: ACACAACCAGGAGTCTCAAC
