# Supplementary material for: The Arabidopsis HY2 Gene Acts as a Positive Regulator of NaCl Signaling during Seed Germination
Source: Int J Mol Sci. 2021 Aug 20;22(16):9009. doi: 10.3390/ijms22169009 (PMC8396667; doi:10.3390/ijms22169009)
Supplement: Supplementary file 1 [file ijms-22-09009-s001.zip › supplymental file.pdf]

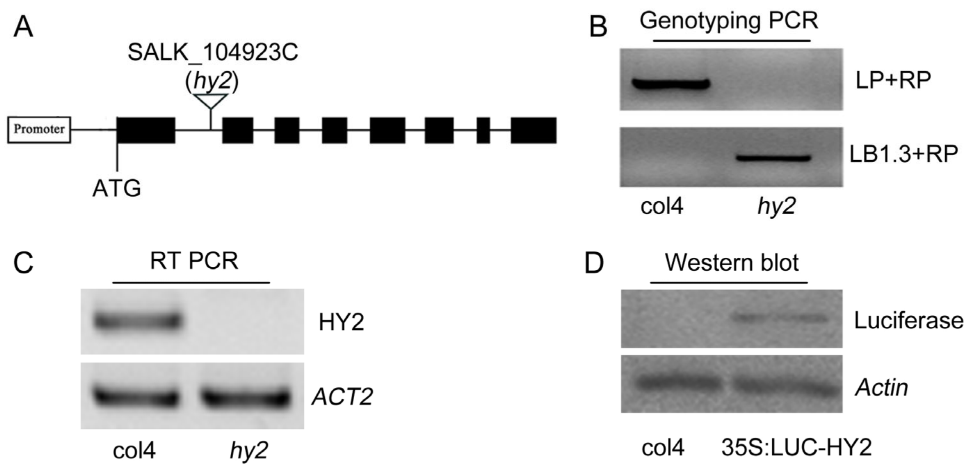

Figure S1. Identification of *LUC-HY2* overexpressing line and *hy2* mutant. (A) Structure diagram of *hy2* (SALK_104923C). **(B)** Genotyping PCR of col4 and *hy2* mutant using specific primers. **(C)** RT-PCR of col4 and *hy2* mutant using specific primers. *ACT2* was used as an internal reference. **(D)** Western blot of col4 and *LUC-HY2* overexpressing line. *Actin* was used as a loading control.

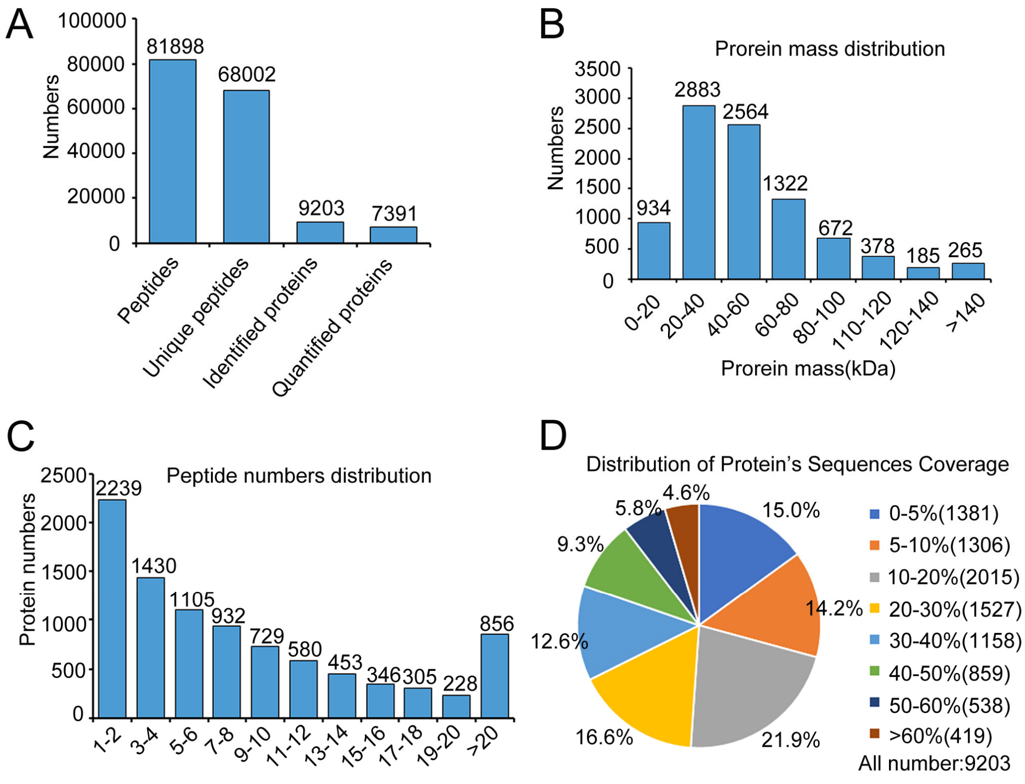

Figure S2. Display of the main data of proteomics. (A) The number of peptides and proteins

identified in proteomics by searching *Arabidopsis* Araport11. **(B)** The size distribution of the identified proteins. **(C)** The proportional distribution of the number of identified peptides. **(D)** The distribution of protein sequence coverage.

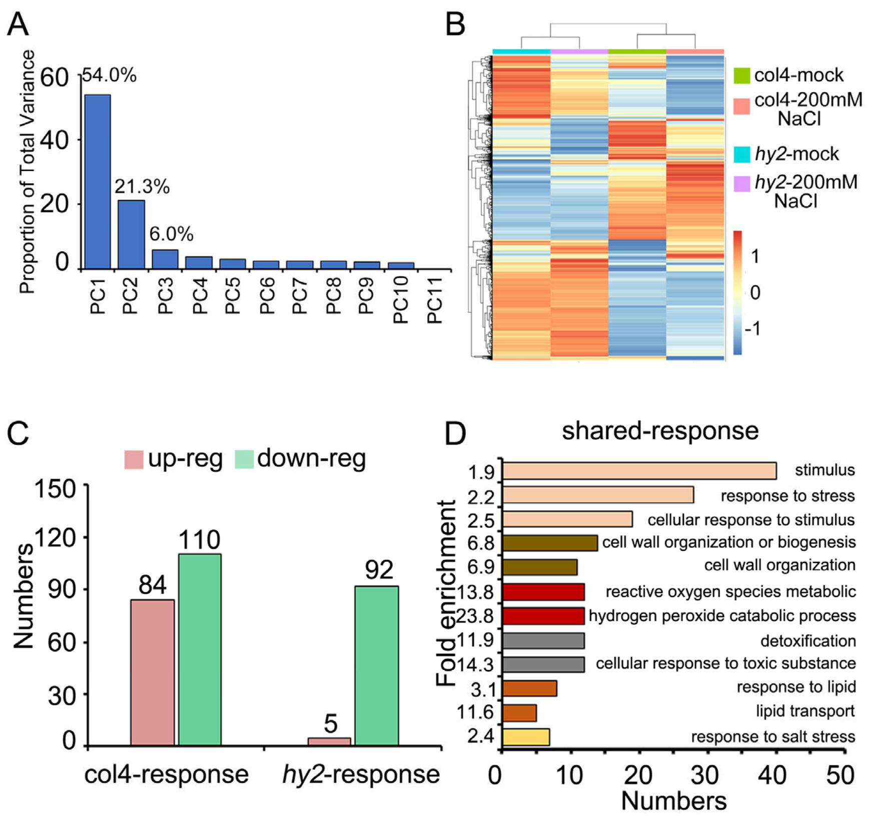

Figure S3. Quantitative proteomics analysis. (A) The proportion of the total variance of each principal component. **(B)** Overall expression level of col4 and *hy2* mutant under salt stress. **(C)** The number of responsive proteins from col4 and *hy2* mutant under salt stress. The red and green squares represent up-regulated and down-regulated proteins, respectively. **(D)** The GO enrichment analysis of col4 and *hy2* mutant shared response proteins under salt stress.

Supplemental Table S1: Basic profiles of proteomics data.

Supplemental Table S2: The data of col4 and *hy2* shared quantified proteins under salt stress.

Supplemental Table S3: The response proteins of col4 under salt stress.

Supplemental Table S4: The response proteins of *hy2* mutant under salt stress.

Supplemental Table S5: GO enrichment analysis of col4 specific quantified and response

proteins under salt stress.

Supplemental Table S6: GO enrichment analysis of *hy2* specific quantified and response proteins under salt stress.

Supplemental Table S7: GO enrichment analysis of col4 specific response proteins under salt stress.

Supplemental Table S8: GO enrichment analysis of *hy2* specific response proteins under salt stress.

Supplemental Table S9: GO enrichment analysis of col4 and *hy2* shared response proteins under salt stress.

Supplemental Table S10: Primer sequences.
